# Multiple‐role mentoring: mentors’ conceptualisations, enactments and role conflicts

**DOI:** 10.1111/medu.13811

**Published:** 2019-02-05

**Authors:** Stephanie N E Meeuwissen, Renée E Stalmeijer, Marjan Govaerts

**Affiliations:** ^1^ Department of Educational Development and Research Faculty of Health Medicine and Life Sciences Maastricht University Maastricht the Netherlands

## Abstract

**Introduction:**

Outcome‐based approaches to education and the inherent emphasis on programmatic assessment in particular, require models of mentoring in which mentors fulfil dual roles: coach and assessor. Fulfilling multiple roles could result in role confusion or even role conflicts, both of which may affect mentoring processes and outcomes. In this study, we explored how mentors conceptualise and enact their role in a multiple‐role mentoring system and to what extent they experience role conflicts.

**Methods:**

We conducted a constructivist grounded theory study at one undergraduate medical school. A purposive sample of 12 physician‐mentors active in a programmatic assessment system was interviewed. Data analysis followed stages of open, axial and selective coding through which themes were constructed.

**Results:**

Three predominant mentoring approaches were constructed: (i) empowering (a reflective and holistic approach to student development); (ii) checking (an observant approach to check whether formal requirements are met), and (iii) directing (an authoritative approach to guide students’ professional development). Each approach encompassed a corresponding type of mentor‐mentee relationship: (i) partnership; (ii) instrumental, and (iii) faculty‐centred. Furthermore, mentors’ strategies, focus, agency provided to students and perception of the assessment system characterised mentoring approaches and relationships. Role conflicts were mainly experienced by mentors with a directing mentoring approach. They used various coping mechanisms, including deviation from assessment guidelines.

**Conclusions:**

In multiple‐role mentoring in the context of programmatic assessment, mentors adopted certain predominant mentoring approaches, which were characterised by different strategies for mentoring and resulted in different mentor–mentee relationships. Multiple‐role mentoring does not necessarily result in role conflict. Mentors who do experience role conflict seem to favour the directing approach, which is most at odds with key principles of competency‐based education and programmatic assessment. These findings build upon existing mentoring literature and offer practical suggestions for faculty development regarding approaches to mentoring in programmatic assessment systems.

## Abstract 2

While serving as both coach & assessor might seem rife with conflict, Meeuwissen et al. report that one's approach to mentoring may determine the likelihood of difficulty.

## Introduction

The importance of mentoring within higher education and especially health professions education has been widely acknowledged.^1^, ^2^, ^3^ Research findings show that effective mentoring programmes positively impact personal, educational and professional outcomes: overall personal well‐being, workplace‐based learning, career decisions and success, as well as job satisfaction and productivity, have been demonstrated to be beneficially affected by mentoring.^1^, ^3^, ^4^, ^5^ Traditionally, definitions of mentoring have focused on: (i) supporting mentees’ development and learning, and (ii) providing a non‐judgemental mentor–mentee relationship and thus a safe environment for mentees.^1^, ^6^ However, definitions and conceptualisations of mentoring seem to be changing in light of the introduction of longitudinal and programmatic assessment (PA) approaches.^7^, ^8^, ^9^

Programmatic assessment aims to support the evaluation of students’ development of competence over time and across various contexts and is characterised by the integration of formative and summative assessment functions, supported through use of longitudinal assessment, portfolios and mentorships.^9^ In PA, portfolios are used to collect cycles of feedback and learning outcomes aimed at guiding individual learning processes as well as supporting summative decisions about student performance.^10^, ^11^ The role of the mentor is essential to the success of PA: mentors can engage mentees in meaningful, reflective dialogues, provide counselling and support the use of follow‐up and feedback.^10^ The mentor is thus tasked with coaching students and stimulating the development of their competences through use of the performance data in the portfolio.^12^, ^13^ However, in PA, the mentor is also asked to advise an independent assessment committee on the students’ progress and competency level. This has resulted in new models of mentoring in which mentors have to fulfil the dual and potentially conflicting roles of coach and assessor.^14^, ^15^

The literature on both mentoring and PA, however, consistently recommends keeping the roles of coach and assessor separate,^1^, ^10^, ^16^, ^17^ as combining these roles may have a negative impact on the mentee, the mentor and the intended outcomes of the mentoring programme.^18^, ^19^ Cavalcanti and Detsky,^19^ for example, stated that mentees might be reluctant to seek help or show their weaknesses to a mentor who is also involved in assessment of their competence. Consequently, mentees may miss important opportunities for improvement of performance. Similarly, findings from the review by Buddeberg‐Fischer and Herta^18^ indicated that constant checking by mentors resulted in high levels of student anxiety. From the mentor’s perspective, potential conflicts of interest may arise from having a vested interest in the mentee's success.^19^, ^20^ Like clinical supervisors, mentors may feel reluctant to ‘fail’ their students, potentially resulting in discrepancies between their personal judgements of students’ performance and the students’ final evaluation.^21^ Earlier research also indicated a strong threat of relational problems between students and mentors when mentors involved in their mentees’ assessments were forced to disclose confidential information or had to break bad news.^22^ Finally, in a study by Bray and Nettleton,^23^ assessment was identified as having less of a priority than imparting clinical knowledge and skills or focusing on the pastoral aspects of mentoring. Having to take on the assessor role caused uncertainty and confusion regarding the expectations of the mentor role.^23^, ^24^

Given the rise of PA within medical education, which has created a need for new models of mentoring, we set out to investigate faculty members’ perceptions of their multiple‐role mentoring within a PA context. More specifically, we used a constructivist grounded theory (CGT) approach and conducted semi‐structured interviews to: (i) explore mentors’ conceptualisation and enactment of their role, and (ii) understand the extent to which they experience role conflicts in a multiple‐role mentoring system embedded in an undergraduate medical curriculum.

## Methods

### Setting

The study was conducted within the undergraduate Master in Medicine (MiM) programme at Maastricht University, the Netherlands. In 2013, PA was introduced as one of the key features of the MiM programme. The MiM comprises 3 years of clinical rotations and is designed according to principles of competency‐based education and assessment, using the Canadian Medical Education Directives for Specialists (CanMEDS) roles as its overarching framework.^25^ PA is supported by a digital portfolio system in which students gather assessment data (e.g. feedback and grades), reflections and additional information on personal and professional development, enabling longitudinal monitoring and assessment of competence development throughout the MiM. Every student is paired with a physician‐mentor for the duration of the MiM. Student and mentor are expected to meet three to four times each year. The mentor is asked to both coach students and support their development of competence, as well as advise an independent assessment committee on the student's level of progress and achievement. This advice is to be based on detailed guidelines, performance standards (milestones) and scoring rubrics supporting interpretation of performance data in the portfolio. For more detailed information, please see Appendix S1.

### Methodology

We used a CGT approach^26^, ^27^ to explore how mentors conceptualise and enact the mentor role and experience possible role conflicts in a multiple‐role mentoring system.^28^ Using CGT, we specifically acknowledge our existing experiential (SNEM) and theoretical (MG and RES) backgrounds in PA and mentoring, which informed data collection and analysis. Data collection and analysis followed an iterative process.^29^ Our results are constructed after interaction with the sampled participants, engagement in the research process and team interpretations of the resulting data.^27^

### Participants

Mentors in the MiM programme were purposively sampled to ensure variety in gender, professional background (surgical specialist, medical specialist or general practitioner), work setting (academic medical centre versus affiliated hospital or practice) and the performance of their mentees (both low‐ and high‐performing students, based on the final decisions made by the assessment committee). All participating mentors had a minimum of 2 years’ experience in the multiple‐role mentoring system and had supervised a minimum of two students during that time. A total of 29 mentors were invited by e‐mail. Out of the 29 mentors, 12 mentors agreed to participate. An overview of participants’ characteristics can be found in Table 1.

**Table 1:** Participants’ characteristics

	Mentors (*n* = 12)
Gender	
Male	4
Female	8
Age	
Mean, years (range)	51 (42–61)
Clinical background	
Surgical specialist	4
Medical specialist	4
General practitioner	4
Work setting	
Academic medical centre	5
Affiliated hospital or practice	7
Attendance training sessions	
No training	1
1–2 training sessions	6
3 (All) training sessions	5

### Data collection

Using semi‐structured interviews, we explored mentors’: (i) conceptualisation of the mentor role; (ii) specific experiences as a coach and assessor of student progress, and (iii) experiences of combining multiple roles (see Appendix S2 for the initial interview guide). The design of the interview guide was informed by key concepts^27^ of mentoring in a multiple‐role mentoring system^19^, ^20^, ^23^ and programmatic assessment.^10^ For the purpose of this study, we defined ‘role conflict’ as situations in which mentors expressed feeling uncomfortable with their role as assessor of a student's progress alongside being a student's coach in learning and development. Interviews were conducted by the author SNEM, which lasted around 60 minutes each and took place between April and June 2016. SNEM is trained and experienced in behavioural interviewing. Alongside the interviews, SNEM also wrote field notes and reflective memos. After 10 interviews data sufficiency was reached and the research team had gained an adequate understanding of the constructed themes.^27^ We conducted two additional interviews to test the developing theory and sampled two male mentors to have a better representation of the mentor population in our sample. All interviews were audiotaped and transcribed verbatim by a professional transcription service that ensured confidentiality.

### Data analysis

SNEM and MG independently read and initially coded the first five transcripts line by line.^30^ Using open and axial coding, the initial codes were compared and discussed by SNEM and MG in order to generate themes and sub‐themes.^31^ The analysis and coding of interviews 11 and 12 confirmed the final coding scheme. In the final phase of analysis, all transcripts were taken together for selective coding.^31^ Team discussions between SNEM, RES and MG led to the final presentation of mentoring approaches and possible role conflicts as reported here. Throughout the process of analysis and writing, established assumptions were discussed. atlas.ti 7 (Scientific Software Development GmbH, Berlin, Germany) was used to support data management during analysis.

### Reflexivity

The research team included a final‐year medical student (SNEM), an educationalist scientist with a focus on programme evaluation (RES) and a medical educator focusing on assessment (MG). SNEM conducted all interviews and therefore physicians who were involved in her assessment were excluded. She was explicitly introduced as a final‐year student investigator and tried to evoke a level of trust and sincerity with the explanations given in the information letter and informed consent paper. SNEM had been with her own mentor for 2 years and she did not perceive her mentor to experience any role conflicts. The other research team members (MG and RES) were involved in evaluation and improvement of mentorship and undergraduate medical education in general. This was communicated explicitly to all participants to mediate any reluctance or superficiality that might have affected the participants. It could be argued that mentors felt reluctant to share negative experiences, because mentors knew SNEM was also receiving mentoring within this mentor system at the time of data collection. Mentors could have also felt more open and honest, as SNEM was not involved in the design and implementation of the feedback and assessment system. During the implementation of the PA system staff appeared reluctant to take on the dual role of coach and assessor. However, questions remained regarding how the mentor system worked in practice. At the start of this research, we held no views other than those on the theoretical aims and potential pitfalls of mentoring programmes within PA. Based on the existing literature, we were curious to discover if and when conflicts were experienced in this setting.

### Ethical considerations

This study was approved by the Dutch Association for Medical Education Ethical Review Board (NVMO ERB 621). Participation was voluntary and all participants signed an informed consent letter. Data were anonymised prior to analysis.

## Results

Three predominant mentoring approaches were constructed from the data: (i) empowering; (ii) checking, and (iii) directing. Each mentoring approach was characterised by its own mentor–mentee relationship: (i) partnership; (ii) instrumental, and (iii) faculty‐centred. Four main factors, related to mentors’ personal background, seemed to influence the mentor's approach and mentor–mentee relationships: (i) mentors’ enacted strategies for mentoring; (ii) the main focus in mentoring; (iii) the degree of agency given to students, and (iv) mentors’ perception of the assessment system. The nature of the adopted mentoring approach seemed to be related to whether and to what extent a role conflict was experienced. A summary of these results is presented in Table 2. In the following we present and discuss these findings in more detail.

**Table 2:** Model of mentoring approaches and conflicts of roles in a multiple‐role mentoring system

Predominant mentoring approaches	Mentor–menteerelationship	Characteristic factors				Conflict of roles
		Mentors’ strategies	Primary focus on mentoring	Extent of students’ agency	Perception of the feedback and assessment system	
Empowering	Partnership	Reflective, mirroring student's behaviour	A holistic approach to the development of students’ personal and professional identity	The student is given considerable agency by the mentor	A support in the mentor role	No: different roles are considered to be a surplus
Checking	Instrumental	Observe, ticking boxes	A check of what the assessment programme prescribes and whether performance standards are met	The student is granted full agency by the mentor	A purpose in itself	No: different roles are considered to be a surplus
Directing	Faculty‐centred	Authoritative, telling students what to do	Give direction on what it takes to become and be a doctor	The student is given a low degree of agency, whereas the mentor steers and has a high degree of agency	A defective system that is not trusted	Yes: mentors feel uncomfortable advising on a student's level

### Empowering mentoring approach

In our sample, five mentors seemed to adopt a predominantly ‘empowering’ mentoring approach. Mentors with this approach aimed to develop a shared understanding and agreement with students on the directions of their learning, similar to working in a partnership. This empowering approach was characterised by a reflective strategy for students’ development, meaning that all feedback, test results and personal issues were mirrored back to the student: mentors with an empowering approach did not provide answers, but asked questions instead. One mentor voiced the enactment as follows:

But I also ask how they see themselves as a person. “Who are you?” “Do you have any idea what kind of doctor you want to be and have you ever thought about that?” (Participant 11)

With mentors who were empowering, students were in the lead and stimulated to set their own goals, select strategies to achieve these goals and monitor their own performance and development. Mentors who were empowering aimed for their mentees to reach their full potential through stimulating the development of lifelong learning skills and supporting students’ individual learning trajectories for the development of their professional identity.

I really try to make that student owner of, which I think is my most important task, his own learning process, and he should use everything for that. (Participant 2)

The empowering approach was maintained even in discussing problematic situations students had experienced.

I had someone [a mentee‐student] who experienced a conflict in the work place. Well, in that case, I ask “What does that mean?” “Where does it come from?” “What does it say about you?” “How could you handle it in a professional way?” (Participant 1)

Mentors with an empowering approach explained how they applied principles of shared decision making in their mentoring, sometimes influenced by the core values of their professional background (e.g. as a general practitioner [GP]).

Well, they [students] have to come up with the solution themselves, but I try to guide them in that, steer them a bit. I am a GP, so I always try to ask thorough questions and consider the context. And of course, as a GP, I have always tried to let patients solve the issue themselves, within their competences. And that is what I also try to do with these students. (Participant 5)

Although these mentors gave students a great degree of autonomy in their learning process, they monitored students’ development by following the offered guidelines for performance assessment. The digital portfolio was seen as a unique tool with entrusted information that empowered each student to develop optimally, both personally and professionally. Meanwhile, the instructions and detailed guidelines on what and how to assess, guided mentors with an empowering approach in interpreting and evaluating the evidence in the digital portfolio.

### Checking mentoring approach

The second mentoring approach that was constructed from the data was an approach best described as ‘checking’. The five mentors in our sample with a predominantly checking mentoring approach fulfilled their role by: (i) monitoring and judging students’ competence development against performance standards, and (ii) identifying students’ problems, and weaknesses in knowledge, skills or professional development to ensure students focused on these issues. This approach led to a mentor–mentee relationship that was largely instrumental, and aimed to ensure that students met all the required performance standards at graduation:

I obediently look at what I have to do. […] So I say well, let's have a look at the list and see which requirements we should meet. (Participant 10)

These mentors felt responsible for detecting problems that would otherwise go unnoticed.

My task towards students? Guarding the red line. Like “is your feedback bound to one internship or does it come back in different internships? Is there a trend, do things come back?” If so, is that a problem and can or should something be done about it? (Participant 6)

Checking mentors did not mention any involvement in students’ personal development. Rather, they seemed to keep a relational distance, requiring students to take full responsibility for their learning and development. They stated that the rules and guidelines of the assessment system determined their mentoring approach and that they based their assessment advice on the information in the portfolio.

It [mentor's assessment advice] is not without obligations. It's stated there. And that's what I say, you know, I don't really have to do much. I just have to read what's in the digital portfolio. (Participant 8)

### Directing mentoring approach

A third group of mentors adopted a predominantly ‘directing’ approach, which was characterised by a faculty‐centred relationship. Compared to empowering mentors, the two directing mentors in our sample acted more authoritatively and seemed to instruct students on which steps to take and how to handle situations. To exemplify the enactment of this mentoring strategy, one mentor told a mentee‐student:

Well, you know what you [mentee‐student] do? […] discuss with your supervising resident, “I want to know everything about inguinal hernias …” And then you let yourself be assessed on that within 2 weeks time. (Participant 3)

These mentors seemed to have a vested personal interest in their student‐mentees and focused on preparing their students for the ‘harsh reality’ of a physician's working life, framed by their personal work experiences in the clinical setting and their personal beliefs on what constitutes a good doctor and what a doctor should be able to do. For example, one mentor working in a peripheral hospital predicted a tough future for current students:

You work in a complex setting, a hospital … but also with other disciplines. Often there are problems and you have to be able to deal with those, and you need to learn that. (Participant 4)

These mentors felt responsible for the performance of students, with the underlying motivation to maintain the professional standards of being a physician in their domain. They were aware of and explicitly took into account strategic behaviours in the context of workplace‐based assessment and learning both from supervisors and students.

… It is a little bit about how clever you [the mentee] are. Meaning, if you know which judgements you need, your portfolio feedback will state “this is done very well, you are assertive”. And if you go to the right people, in the right way, with the right [way of asking them for feedback] … (Participant 3)

Therefore, the directing mentors in our sample seemed to lack trust in the assessment system and the usefulness of feedback in the portfolio, and they preferred actually seeing their mentees function in daily practice, so that they themselves could form an opinion about the mentees’ performance.

I am only able to evaluate students when I had the opportunity to work with them. […] and I am less able to think something about it [students’ ability to be a doctor] because I am looking at all these Mini‐CEXs [Mini‐Clinical Evaluation Exercises], where narratives are merely left blank. Well, what can you do then? (Participant 4)

### Experiences of role conflicts

The predominantly empowering and checking mentors perceived the combination of the assessor and coach roles as added value for the student's development and felt that they were facilitated by the feedback and assessment system in performing both roles. Although differing in approach, empowering and checking mentors perceived assessment to be embedded in the coaching process, as their coaching entailed having regular feedback conversations with the student, looking back on past performance and defining actions to close the gap between current performance and goals to be achieved.

It supports me if someone says “yes I can see my test results or these domains are below requirements or I should do my communication differently”. Yes, nice, then we [mentor and mentee] see it together. I don't think the next step [the assessment] is threatening my mentor role. If I then say “well then you have points of attention there”. (Participant 2)

Overall, these mentors felt confident in making judgements of students’ performance based on the aggregated evidence gathered in the digital portfolio. They further explained that they expected self‐directedness and self‐assessment of their students in filling the portfolio and awareness of performance standards. To conclude, the roles of coach and assessor were seen as integral to the mentor role of empowering and checking mentors:

I think that by judging them [students], you are already coaching them. That if something has to be remediated by the student that you explain “ok, this is the process through which you need to learn”. I think that it is added value [of the role]. (Participant 12)

Role conflicts seemed more likely to be experienced by mentors with a predominantly directing approach. These mentors stated that the assessor role as a whole was an unpleasant experience, where providing assessment advice was seen as disrupting their task as coach and creating distance in the mentor–mentee relationship.

Looking at myself, I think if someone judges you, then your relation with a mentee is rather a teacher‐apprenticeship relationship and not a relation in a sense that you [as a student] are going to tell how your private life is going, which affects your training in a negative way. In my opinion, that [being a coach and assessor] does not work well together. (Participant 4)

Also, these mentors’ performance standards did not always meet the performance standards as prescribed by the assessment system. Instead, they had strong individual beliefs on what was important for students. They thus built upon their own experiences and preferred to make judgements based on direct observations. They mistrusted ‘second‐hand information’ in the students’ portfolio, which they regarded as low in credibility:

I have never seen the student [my mentee] in a white coat. And it's possible that he is making a mess of it and everyone else is thinking “wow what is he doing here in the hospital?” But I am not able to see that. (Participant 3)

The directing mentors in our sample were worried that negative advice could have a negative impact on the student's development and future career. From the perspective of theory of assessment, they kept seeing assessment as a summative process (assessment of learning) and not a formative process (assessment for learning). These mentors’ feelings of responsibility and having a vested interest in students’ professional development were reflected in specific assessment behaviours such as failing to document relevant personal information in the portfolio:

Let me be honest, with that guy [my mentee‐student], I did not put that [negative comments] in his portfolio. […] I said “you know, I am just telling you this now. And I am not going to put this in your portfolio, because it might haunt you”. So I like to give him some personal information now and then, “be aware of this and in your assessment, be aware of that”. (Participant 3)

These mentors used different additional mechanisms to cope with their experienced role conflict. They refrained from making advisory judgements themselves and heavily relied on student's self‐assessment. In addition, the conversations during mentor meetings became a source of assessment rather than the evidence in the portfolio itself. The portfolio was then passed on to others (i.e. the portfolio assessment committee) to review the evidence instead of doing it themselves.

With this one case I actually let the student decide herself. If it would have been expected from me to make that decision, whether she should continue in that case or not, I would find that extremely uncomfortable. (Participant 3)

## Discussion

We undertook this study to explore mentors’ conceptualisation and enactment of their role in a multiple‐role mentoring system and the extent to which mentors experience role conflicts. Mentors’ role conceptualisations and enactments were reflected in three predominant mentoring approaches and, in parallel, three mentor–mentee relationships: *empowering* mentors worked together with their mentee‐students in *partnership*,* checking* mentors developed more *instrumental* mentor–mentee relationships, whereas *directing* mentors typically developed more *faculty*‐*centred* working relationships with their mentees. Each of the approaches was characterised by a preference for certain mentor strategies, the focus of mentoring, perception of the feedback and assessment system, and the level of agency in mentor–mentee relationships. The extent to which a conflict of roles was experienced, seemed to be related to mentors’ preferred mentoring approach: in our study role conflicts were experienced by mentors adhering to a more directing mentoring approach. Notably, our study shows that incorporating elements of assessment in definitions of mentoring does not need to be problematic.

The adopted mentoring approach seemed to be associated with mentors’ professional background and work setting. As such, mentors’ conceptualisations and enactments seemed to be either informed by demands of their current work settings (e.g. directing mentors felt very committed to developing students into professionals that are truly ‘fit for actual practice’) or similar to their behaviour towards patients (e.g. empowering mentors predominantly applied models of shared decision making such as used in general practice^32^). Additionally, deeply rooted beliefs about learning and (dis)trust in the assessment system influenced mentoring approaches. For example, the directing mentors in our sample seemed to be driven by assessment *of* learning, instead of assessment *for* learning priciples.^9^

In her study on mentoring in university communication and journalism departments, Buell^33^ described four different mentoring models (cloning, apprentice, nurturing and friendship), which to a large extent resemble findings from our study (cloning – directive, apprentice – checking, nurturing – empowering). Contrary to Buell’ s findings, our participants did not seem to adopt the friendship model – an approach in which the mentor–mentee relationship is considered to be a personal friendship.^33^ Rather, the mentors in our study all seemed to develop and maintain a professional relationship. Our study moves beyond the findings of Buell by describing both characterising mentor–mentee relationships and factors that influence the enactment of multiple‐role mentoring in medical education. An important nuance in our findings might be the concepts of different or evolving mentoring approaches. DeCastro et al.^34^ highlighted how individual mentors may adopt different mentoring roles depending on the relationship with the particular mentee‐student in the context of medicine. Likewise, Buell^33^ described that mentoring models could take place in a continuum of mentor–mentee interactions. Furthermore, she suggested that the ultimate relationship with mentees came from combined aspects of different mentoring models.^33^ It has also been previously stated that as the mentee develops, the role of the mentor evolves.^35^ The predominant mentoring approaches and relationships identified in our study were at the heart of participants’ mentoring but could be accompanied by characteristics from one or both of the other approaches. The mentoring approaches as constructed from our data should therefore be the focus of further (longitudinal) research to examine whether the combination and development of mentoring approaches also holds within the setting of PA.

In contrast to previous literature,^1^, ^10^, ^16^, ^17^ our findings show that multiple‐role mentoring systems are not necessarily associated with role conflicts. Whereas Heeneman et al.^15^ linked role conflicts to a lack of mentor experience, the personal relationship with the mentee and uncertainties about students’ end level, our results point more towards the influence of the mentoring approach, which in turn depends on a complex interplay of individual mentor factors. Our finding that a more directing mentoring approach, linked to a faculty‐centred relationship, is more strongly related to the negative experiences of mentors, does align with the findings by Arntfield et al.^36^ They emphasised the importance of student agency and a ‘bi‐directional and cyclical’ process to achieve student–mentor engagement and feedforward in positive experiences.^36^ It is noteworthy that the importance of student agency has been voiced by students themselves as it helps them learn to take control of their learning and assessment experience.^37^

The mentors who experienced role conflict in our study described several coping mechanisms. These mechanisms were partly related to a lack of trust in programmatic, workplace‐based assessment and having to judge ‘second‐hand information’. Assessors wanting to rely on their own observations were previously reported by Hawe^38^ and Driessen and Scheele,^39^ who described how assessors were ‘intuitive’ and wanted to trust their ‘gut feelings’ instead of using the information in a portfolio. The need for faculty members to identify and internalise set standards for them to comfortably work with them is consistent with the work of Govaerts et al.^40^ Our study, however, clearly points out how deeply rooted personal beliefs about what a mentor should be and do may hinder adoption and use of external guidelines and performance standards.

Notably, our study shows that moving on from the old definition and conceptualisation of mentoring – supporting mentees’ development and providing a non‐judgmental mentor–mentee relationship^1^, ^6^ – does not need to be problematic. Whereas the old definition of mentoring incorporated the non‐judgemental aspect,^6^, ^10^ both the empowering and checking mentoring approaches are in line with current developments in medical education in which the assessment process is seen as a personal educational plan‐do‐study‐act cycle, informed by ongoing formative assessments to guide learning, improvement and goal achievement.^41^ Additionally, the empowering approach seemed to explicitly focus on enhancing student's self‐regulation and agency in the learning and development process. Hence, this study shows that a multiple‐role mentoring system in PA can lead to ongoing performance improvement and a learning culture in which assessment is a source of insight, integrated with the learning process.^42^

### Limitations

The findings from our study should be interpreted in light of certain limitations. First, this study involved only one Dutch medical school, which implemented PA in the clinical training programme. Little can be said about transferability of our findings to other (inter)national settings where mentors’ contextual and personal factors may be influenced by (different) cultural aspects. However, every attempt was made to augment transferability by providing a thick description^29^ of the context in which data were collected. Additionally, the short duration of the new Master's curriculum may have limited mentors’ experience in fulfilling their mentor role and the assessor role in particular. Nevertheless, our data clearly showed a range of perspectives on the assessor role. With the use of behavioural interviewing, the questions aimed to unravel past experiences and behaviour in mentoring. We cannot know, however, to what extent our data reflect actual practice (i.e. what mentors actually do).

### Implications for practice and future research

Our findings on the conceptualisation and enactment of the mentor role and the experience of role conflicts provide important information that can be used to optimise mentor training and coaching. Faculty development for mentors in PA systems should not only focus on key principles underpinning PA, portfolio‐based learning and assessment, but also on mentor beliefs and how these may affect the interaction between mentoring approach, mentor–mentee relationships and potential outcome of mentorships.

Because our results did not follow‐up on mentors' practices, there could be more nuances in the mentoring approaches that we found. Future research should explore the development of mentors and mentoring approaches over time. Additionally, exploring whether, why and how mentors use different mentoring approaches seems worthwhile. Furthermore, how certain mentoring approaches impact student learning, performance and experiences is of interest. Observational research would provide valuable additional insights into what mentors actually do in practice and how mentoring develops over time.

## Conclusions

Mentoring in multiple‐role mentoring systems appears to be a balancing act but does not necessarily result in role conflict. With the empowering mentoring approach, a learning culture can be developed in which assessment is integrated with the learning process. The proposed mentoring approaches and coping mechanisms represent a starting point for further research and offers practical implications for optimally designing and developing mentoring programmes in curricula working with PA.

## Contributors

all authors (SNEM, RES and MG) contributed substantially to the conception and design of the study. Data was collected by SNEM. Initial coding was performed by SNEM and MG. RES joined for selective coding. SNEM drafted the initial manuscript, which was critically revised by all authors (SNEM, RES and MG). All authors (SNEM, RES and MG) approved the final manuscript for submission.

## Funding

None.

## Conflicts of interest

the authors (SNEM, RES and MG) declare that they have no competing interests.

## Ethical approval

Ethical approval was obtained from the Dutch Association for Medical Education Ethical Review Board (NVMO ERB 621).

## Supporting information


**Appendix S1.** Requirements for mentors and positioning of mentor judgements.
**Appendix S2.** Original interview guide.
