# Supplementary material for: Multiple‐role mentoring: mentors’ conceptualisations, enactments and role conflicts
Source: Med Educ. 2019 Feb 5;53(6):605–15. doi: 10.1111/medu.13811 (PMC6590242; doi:10.1111/medu.13811)
Supplement: Supplementary file 1 — Appendix S1. Requirements for mentors and positioning of mentor judgements. Appendix S2. Original interview guide. [file MEDU-53-605-s001.docx]

*Appendix 1 – Requirements for mentors and positioning of mentor judgements*

| **Requirements:** mentors are medical doctors, working as physicians in a university hospital, affiliated hospital, general practice, or other health care institutions. Medical departments are requested to recruit a number of mentors in their department. The medical departments are financially compensated for a set number of hours for each mentor. Mentors are offered multiple voluntary training sessions, including orientation to the assessment programme and mentoring system, clarification of the mentoring role, discussion of guidelines and training in skills relevant to coaching and assessment.  During mentor meetings, the information in the portfolio including the student’s self-assessment is discussed to longitudinally: a) support competence development by guiding the student’s:  i) self-assessment,   ii) monitoring of competency development  iii) setting of new learning goals b) advise on the assessment of the student’s progress towards predefined performance standards in the annual performance assessment meetings.    **Positioning mentor judgements:** students and mentors are provided with specific guidelines to guide low and high stakes assessment moments. The mentor’s recommendation on the student’s progress and competency level is forwarded to an independent portfolio assessment committee as advisory. This recommendation, however, plays a leading role in the formal and summative decision made by the portfolio assessment committee. |
| --- |

*Appendix 2 - Original interview guide*

1. How do see as your role as mentor?

2. What do you feel as an important part of your role as mentor?

3. What is your perception of the role as coach?

4. What is your perception of the role as assessor?

5. What kind of questions or issues do mentee-students come up with?

6. What is your experience regarding the combination of roles as coach and assessor?

7. Which situation can be named as an example where you were fully aware of the combination of roles?

8. What is your experience of the combination of roles regarding the effects on students?

9. In case you experienced a role conflict: how did you proceed in your role?

10. Which factors influence implementation of the combination of roles?

11. What is your perception of the combination of roles to the ultimately realized mentor role?

12. How do you value the current mentoring system regarding students’ competence development?

13. How do you value the current mentoring system regarding the experienced relationship with mentee-students?

14. What would you recommend to change in the current mentoring system?
